# Screening of premature ovarian insufficiency associated genes in Hungarian patients with next generation sequencing

**DOI:** 10.1186/s12920-024-01873-z

**Published:** 2024-04-22

**Authors:** Anett Illés, Henriett Pikó, Kristóf Árvai, Veronika Donka, Olívia Szepesi, János Kósa, Péter Lakatos, Artúr Beke

**Affiliations:** 1 Department of Internal Medicine and Oncology, Semmelweis University, Budapest, Hungary; 2 Department of Obstetrics and Gynecology, Semmelweis University, Budapest, Hungary

**Keywords:** Premature ovarian insufficiency, POI, POF, Infertility, Hungarian, NGS, Next generation sequencing

## Abstract

**Background:**

Premature ovarian insuffiency (POI) is one of the main cause behind infertility. The genetic analysis of POI should be part of the clinical diagnostics, as several genes have been implicated in the genetic background of it. The aim of our study was to analyse the genetic background of POI in a Hungarian cohort.

**Methods:**

The age of onset was between 15 and 39 years. All patients had the 46,XX karyotype and they were prescreened for the most frequent POI associated FMR1 premutation. To identify genetic alterations next-generation sequencing (NGS) of 31 genes which were previously associated to POI were carried out in 48 unrelated patients from Hungary.

**Results:**

Monogenic defect was identified in 16.7% (8 of 48) and a potential genetic risk factor was found in 29.2% (14 of 48) and susceptible oligogenic effect was described in 12.5% (6 of 48) of women with POI using the customized targeted panel sequencing. The genetic analysis identified 8 heterozygous damaging and 4 potentially damaging variants in POI-associated genes. Further 10 potential genetic risk factors were detected in seven genes, from which EIF2B and GALT were the most frequent. These variants were related to 15 genes: *AIRE*, *ATM*, *DACH2*, *DAZL*, *EIF2B2*, *EIF2B4*, *FMR1*, *GALT*, *GDF9*, *HS6ST2*, *LHCGR*, *NOBOX*, *POLG*, *USP9X* and *XPNPEP2*. In six cases, two or three coexisting damaging mutations and risk variants were identified.

**Conclusions:**

POI is characterized by heterogenous phenotypic features with complex genetic background that contains increasing number of genes. Deleterious variants, which were detected in our cohort, related to gonadal development (oogenesis and folliculogenesis), meiosis and DNA repair, hormonal signaling, immune function, and metabolism which were previously associated with the POI phenotype. This is the first genetic epidemiology study targeting POI associated genes in Hungary. The frequency of variants in different POI associated genes were similar to the literature, except *EIF2B* and *GALT*. Both of these genes potential risk factor were detected which could influence the phenotype, although it is unlikely that they can be responsible for the development of the disease by themselves. Advances of sequencing technologies make it possible to aid diagnostics of POI Since individual patients show high phenotypic variance because of the complex network controlling human folliculogenesis. Comprehensive NGS screening by widening the scope to genes which were previously linked to infertility may facilitate more accurate, quicker and cheaper genetic diagnoses for POI. The investigation of patient’s genotype could support clinical decision-making process and pave the way for future clinical trials and therapies.

## Background

Premature ovarian insufficiency (POI) or premature ovarian failure (POF) is the cessation of ovarian function before 40 years of age. At the end of the 2000s years, the phrase primary ovarian insufficiency (POI) was considered to better describe this premature-ovarian-aging condition, highlighting that women with this dysfunction sometimes spontaneously have follicular development and/or returned menses and/or conceive after the diagnosis is made [1, 2]. POI is defined by the depletion of ovarian follicles, leading to infertility before the age of 40 years with a wide range of clinical phenotypes [3]. This condition is characterized by the cessation of menses (amenorrhea or oligomenorrhea) for at least 4 months, increased gonadotropin levels (FSH > LH), and hypoestrogenism [4]. The first red flag can be the primary amenorrhea, which is usually diagnosed at a young age in individuals with delayed puberty and an absence of breast development and menarche. However, secondary amenorrhea is the most frequent POI phenotype which is manifested at an age from 20 to 40 years and is defined by normal pubertal development and an irregular menstrual cycle followed by amenorrhea [3]. Despite the incomplete epidemiological data, the prevalence of POI appears to related with ethnicity and increase with age (1:10 000 by age 20, 1:1000 by age 30, and 1:100 by age 40) [5].

POI can be caused by genetic defects, autoimmune diseases, iatrogenic factors (chemotherapy or radiation therapy), viral infections, toxins, or it can remain idiopathic despite exhaustive investigation [5]. However, the observation that many affected women have positive family history increase the probability of inherited POI [6]. Regarding genetic causatives, both chromosomal abnormalities and monogenic defects can result POI phenotype. After confirmation of POI clinical diagnosis, chromosomal analysis, fragile-X premutation (*FMR1*) analysis, adrenal (21-hydroxylase) and thyroid antibody assessment, as well as pelvic ultrasonography should be performed [3]. Frequency of chromosomal abnormalities is approximately 10–13% [7]. Evaluation of karyotypes for numerical changes can be performed by cytogenetic analysis, and different approaches, such as aCGH have evolved to identify copy number variations (CNVs) in the background of POI [8]. Moreover, syndromic POI may also be caused by the expansion of a CGG repeat in the 5’ regulatory region of the *FMR1* gene, which causes Fragile-X syndrome. Thus, the presence of *FMR1* premutation should be investigated in women with POI, since this is associated with POI in approximately 20% of carrier women [9]. Further studies suggested that microdeletions within *FMR2* gene may also be a significant cause of POI [10]. This screening might be helpful for the identification of POI etiology, even so most POI cases remain without a clarified background. Thus, if the previously mentioned examinations have no positive results which can confirm POI diagnosis monogenic analysis should be the next step.

Since POI syndrome show high phenotypic variance with a heterogeneous genetic aetiology because of the complex network controlling human folliculogenesis NGS analysis may facilitate more accurate, quicker and cheaper genetic diagnoses for POI. Furthermore, an oligogenic etiology for this disorder has been proposed which also highlight the necessity of multigene panel sequencing [11, 12]. Previous evidence suggested that these genes might be clustered on the female sex chromosome in the POF1 and POF2 loci [13]. There are several X and autosomal encoded genes which play a potential role in human folliculogenesis, thus they are suggested to be prominent POI candidate genes. By further investigating their functional contribution to the genetic etiology of POI in the clinic, a better diagnostic pipeline could be generated.

In the NGS era, our knowledge about the molecular basis of idiopathic POI had rapidly expanded. High-throughput sequencing techniques have described several novel pathogenic variants of well-known genes (*FSHR, GDF9, BMP15, FIGLA*, and *NOBOX*) in association with POI [3]. These genes were suggested to contribute to POI etiology because of their roles in germ cell development, meiosis and DNA repair, oogenesis, folliculogenesis and hormone signaling, metabolism, autoimmune association, and sex chromosome remodelling. Furthermore, the large-scale genetic analysis paves the way to identify further variants in genes which are still unknown in the background of POI. The expansion of our knowledge could increase the positive findings during the genetic analysis of POI patients and could provide new ways to find potential therapies for women with POI. To date, it has been the first comprehensive genetic study on Hungarian patients with POI that exploits the opportunities provided by the NGS method.

## Methods

### Subjects

We recruited 48 unrelated patients from Hungary, who were diagnosed with POI, presented with amenorrhea for at least 6 months before age 40 and had FSH plasma levels above 40 IU/L. The average age of the patients was 35.98 ± 6.34 years, and menopause occurred at an average age of 33.06 ± 6.13 years (min. 15 years, max. 39 years). A total of 38/48 patients had menopause between the ages of 30 and 39 (79.17%), while 7/48 (14.58%) and 3/48 (6.25%) cases were menopause between the ages of 20 and 29 and up to the age of 20, respectively. All patients had the 46,XX karyotype and they were prescreened for the most frequent POI associated *FMR1* premutation. Targeted panel sequencing was performed on 48 POI patients (P01∼P48). Our work complies with the principles laid down in the Declaration of Helsinki. The work has been approved by the ethics committee Medical Research Council (No: 4288-0/2011-EKU). All patients gave informed written consent to participate.

### Targeted panel sequencing

We have aimed to cover all the well-established POF risk loci. The list of the 31 investigated genes was compiled based on literature data.

Amplicon library was prepared using the Ion AmpliSeq Library Kit Plus combined with the (ThermoFisher, MA, USA). Briefly, 10 ng of genomic DNA was added to multiplexed primer pairs (2 pools) and amplified with the following PCR conditions: at 99 °C for 2 min; at 99 °C for 15 s and at 60 °C for 4 min (19 cycles) and holding at 10 °C. Primers were partially digested using a FuPa reagent, and then sequencing adapters and barcodes were ligated to the amplicons. The library was purified using the Agencourt AMPure XP Reagent (Beckmann Coulter, CA, USA). The concentration of the final library was determined by Ion Library TaqMan Quantitation Kit (ThermoFisher, MA, USA) on an ABI 7500 qPCR instrument with absolute quantification method. Template preparation was performed with Ion 520 OT2 Kit (ThermoFisher, MA, USA) on semi-automated Ion OneTouch 2 instrument using emPCR method. After breaking the emulsion, the non-templated beads were removed from the solution during the semiautomated enrichment process on Ion OneTouch ES (ThermoFisher, MA, USA) machine. After adding the sequencing primer and polymerase, the fully prepared Ion Sphere Particles (ISPs) were loaded into an Ion 520 chip, and the sequencing runs were performed using the Ion S5 Sequencing kit (ThermoFisher, MA, USA) with 500 flows.

Sequence data from the Ion Torrent run were analyzed using the platform-specific pipeline software Torrent Suite v5.10 for base calling, trim adapter and primer sequences, filtering out poor quality reads, and demultiplex the reads according to the barcode sequences. Briefly, TMAP algorithm was used to align the reads to the hg19 human reference genome, and then, the variant caller plug-in was executed to search for germline variants in the targeted regions. Integrative Genomics Viewer (IGV) [14] was used for visualization of the mapped reads. Variants were annotated using the Ion Reporter (ThermoFisher, MA, USA) and Varsome [15] software. Variant classification followed the latest ACMG guideline.

## Results

At Semmelweis University Department of Obstetrics and Gynecology Genetic testing for POI was performed in 142 patients. In 12 cases we detected a cytogenetic difference 8.45%. *FMR1* gene variation was detected in 17 cases 11.97% (RP-PCR, CGG repeat). Additional genetic testing was performed in 48 patients, sequencing: Next-generation sequencing (NGS), targeted panel sequencing.

To identify potential causative variants of POI by using targeted panel sequencing, we analyzed DNA samples of 48 unrelated patients with POI. In our cohort two heterozygous potentially pathogenic and 6 heterozygous probably pathogenic alterations, 4 heterozygous VUS (variant with unknown significance) and 10 potential genetic risk factors were identified in 15 POI related genes (Table 1. and Table 2., respectively). In addition, 6 patients carried VUS or risk factor in two different genes which suggested a potential oligogenic effect (Table 2.). Further two variants, similarly to a previous observation [16] were more frequent in our cohort than in the control population, such as *EIF2B4* c.*1C > T in four patients and *HS6ST2* c.146 C > T in 3 patients. Clinical interpretation and indication for the detected variants were thoroughly classified by the aid of VarSome [15] and Franklin (Available online: https://franklin.genoox.com). The age at menarche, the age at POI diagnosis, hormone levels and identified genes carrying potential causative variants are shown in Table 3.

Sequencing of genes previously related to POI resulted to identify 12 potentially damaging variants and 10 potential risk factors, 12/48 (25%) and 17/48 (35.4%) subjects, respectively. All of the identified variants were heterozygous. Most of the identified variants were missense (20 of 22, 90.9%), one was frameshift (1 of 22, 4.5%), and one was a splicing variant (1 of 22, 4.5%). Substitutions in *AIRE* (c.901G > A; p.Val301Met) and *ATM* (c.680 C > T; p.Ser227Leu, c.4424 A > G; Tyr1475Cys) were identified in 3 of 48 patients. Both *ATM* variants associated with potential risk factors in *NOBOX* genes (P24 and P26). P20 carried two coexisting alterations: the c.958G > A; p.Gly320Ser in *GDF9* gene and the c.803G > C; p.Gly268Ala in *POLG* gene, which was also identified in P18. In association with ovarian development *DAZL* (c.863G > A; p.Ser288Asn, c.380G > A; p.Arg127His), *EIF2B2* (c.380 C > T; p.Ala127Val) and *LHCGR* (c.211G > C; p.Gly71Arg) variants were described. In addition, two substitutions were detected in *XPNPEP2* gene (c.828delT; p.Phe276LeufsTer12, c.460G > A; p.Val154Met) in two unrelated patients. The second detected *AIRE* variant (c.1322 C > T; p.Thr441Met) was identified in P08 patient, which is considered as risk factor in case of POI. Two potential risk factors were detected in *DACH2* gene (c.107 C > T; p.Pro36Leu, c.1245G > C; p.Glu415Asp). The previous variant was described in P03, who also carried a substitution in the *USP9X* gene (c.6722T > C; p.Val2241Ala). In two patients (P31 and P40) who carried *EIF2B4* splice site variant also had a second risk factor (*GALT* c.940 A > G; Asn314Asp and *NOBOX* c.218 A > G; His73Arg, respectively). This variant in *GALT* gene was found in 7 other patients. A missense variant in *FMR1* gene in P10 patient suggested to increase the susceptibility to develop POI phenotype.

All of the 15 affected genes previously associated with POI were playing a role in crucial biological processes, such as autoimmune association, meiosis, DNA repair, sex chromosome rearrangement, ovarian development and metabolism.

**Table 1:** Potential causal variants found in 12 POI patients via targeted panel sequencing

Gene	ACMG	dbSNP ID	Sequence change		Type	ClinVar	MAF	Pathogenicity						Patient ID	Reference
			cDNA	AA				P/B	CADD	VEST	SIFT	PolyPhen	GERP++		
*AIRE*	VUS-P (PM2, BS2, PP3)	rs150634562	c.901G > A	V301M	Missense	VUS	< 0.01	22/4	25	0.53	0.02	0.95	3.86	P42	[17]
*ATM*	VUS (PM2, BP4)	rs762998620	c.680 C > T	S227L	Missense	VUS	< 0.01	4/22	23	0.19	0.04	0.01	4.45	**P24**	*
*ATM*	VUS (BP6)	rs34640941	c.4424 A > G	Y1475C	Missense	VUS	< 0.01	9/14	22	0.29	0.09	0.01	5.56	**P26**	*
*DAZL*	VUS (PM2)	-	c.863G > A	S288N	Missense	-	-	11/15	23	0.47	0.03	-	6.06	P09	*
*DAZL*	VUS-LP (PM2)	rs1360420397	c.380G > A	R127H	Missense	-	< 0.01	15/12	25	0.52	0.04	0.70	5.11	P36	*
*EIF2B2*	VUS-LP (PM2, PP2)	rs150617429	c.380 C > G	A127G	Missense	VUS	< 0.01	12/14	22	0.27	0.06	0.01	5.36	P22	[18]
*GDF9*	VUS (PM2, BP4)	rs1321340406	c.958G > A	G320S	Missense	-	< 0.01	3/24	17	0.08	0.31	0.02	3.85	**P20**	*
*LHCGR*	VUS-LP (PM2, PP3)	rs746197082	c.211G > C	G71R	Missense	-	< 0.01	22/6	25	0.78	0.05	1	5.89	P05	[19, 20]
*POLG*	VUS-P (PP2, PP3, PP5, BS2)	rs61752784	c.803G > C	G268A	Missense	VUS	< 0.01	10/2	25	0.99	0	1	5.48	P18, **P20**	[21]
*USP9X*	VUS-LP (PM2)	-	c.6722T > C	V2241A	Missense	-	-	16/9	24	0.72	0	-	5.56	**P03**	*
*XPNPEP2*	VUS-LP (PM2)	-	c.828delT	F276Lfs*12	Frameshift	-	-	1/0	-	0.29	-	-	5.78	P04	*
*XPNPEP2*	VUS-LP (BP1, BP4, PM2)	rs1308764609	c.460G > A	V154M	Missense	-	< 0.01	21/4	22	0.52	0	0.99	4.5	P19	*

VEST analysis generates values between 0 and 1, and scores ≥ 0.5 were classified as pathogenic variants and those < 0.5 as benign. CADD analysis generates a PHRED-like scaled value. Scores ≥ 20 were classified as pathogenic variants and those < 20 as benign. PolyPhen analysis generates values between 0 and 1, and scores 0.0 to 0.15 were predicted to be benign, 0.15 to 0.85 as possibly damaging and 0.85 to 1.0 as damaging. The SIFT score ranges from 0 to 1, and scores 0.0 to 0.05 were considered deleterious and 0.05 to 1.0 as tolerated. GERP + + scores range from − 12.3 to 6.17, with higher scores indicating higher evolutionary constraint. A score greater than 2 considered as constrained. Abbreviations: ACMG: American College of Medical Genetics, cDNA: complementary DNA, AA: amino acid change, MAF: minor allele frequency (gnomAD v3.1.2 non-Finnish (controls/biobanks), P/B: pathogenic/benign prediction.

**Table 2:** Potential susceptibility factors found in 17 POI patients via targeted panel sequencing

Gene	dbSNP ID	Sequence changes		Type	ClinVar	MAF	Pathogenicity						Patients ID	Reference
		cDNA	AA				P/B	CADD	VEST	SIFT	PolyPhen	GERP++		
*AIRE*	rs72650677	c.1322 C > T	T441M	Missense	VUS	< 0.01	6/21	12	0.73	0.02	0.24	0.63	P08	[22]
*DACH2*	rs147377892	c.107 C > T	P36L	Missense	-	< 0.01	11/12	21	0.10	0	0.02	4.49	**P03**	[23]
*DACH2*	rs148179765	c.1245G > C	E415D	Missense	-	< 0.01	7/16	16	0.08	0.3	0.08	1.81	P14	*
*EIF2B4*	rs41288827	c.*1C > T	-	Splice site	B	0.01	0/2	4	-	-	-	-3.26	P11, P12, **P31**, **P40**	[16]
*FMR1*	rs139029212	c.818 A > G	K273R	Missense	VUS	< 0.01	7/16	21	0.25	0.49	1	5.78	P10	[24]
*GALT*	rs2070074	c.940 A > G	N314D	Missense	VUS	0.095	2/19	17	0.77	1	0	5.3	P23, **P26**, P29, **P31**, P32, P44, P45, P48	*
*HS6ST2*	rs181526961	c.146 C > T	S49L	Missense	-	0.01	4/6	24	0.34	0	0.92	3.65	P37, P38, P39	*
*NOBOX*	rs139083352	c.1849 C > T	H617Y	Missense	-	< 0.01	3/20	3	0.18	0.18	0.01	0.48	**P26**	*
*NOBOX*	rs115882574	c.1826 C > T	P609L	Missense	B/LB	< 0.01	9/15	20	0.23	0.06	0.59	3.56	**P24**	*
*NOBOX*	rs373109638	c.218 A > G	H73R	Missense	-	< 0.01	3/23	1	0.19	0.36	0	-3.36	**P40**	*

VEST analysis generates values between 0 and 1, and scores ≥ 0.5 were classified as pathogenic variants and those < 0.5 as benign. CADD analysis generates a PHRED-like scaled value. Scores ≥ 20 were classified as pathogenic variants and those < 20 as benign. PolyPhen analysis generates values between 0 and 1, and scores 0.0 to 0.15 were predicted to be benign, 0.15 to 0.85 as possibly damaging and 0.85 to 1.0 as damaging. The SIFT score ranges from 0 to 1, and scores 0.0 to 0.05 were considered deleterious and 0.05 to 1.0 as tolerated. GERP + + scores range from − 12.3 to 6.17, with higher scores indicating higher evolutionary constraint. A score greater than 2 considered as constrained. Abbreviations: ACMG: American College of Medical Genetics, cDNA: complementary DNA, AA: amino acid change, MAF: minor allele frequency (gnomAD v3.1.2 non-Finnish (controls/biobanks),, P/B: pathogenic/benign prediction.

**Table 3:** Clinical characteristics and molecular findings of POI patients carrying potential causative variants

Patients ID	Menarche age (year)	POI onset age (year)	Hormone values		Gene
			FSH (lU/L)	LH (ng/mL)	
P03	13	34	89,53	0,010	*USP9X, DACH2*
P04	12	34	98,19	0,016	*XPNPEP2*
P05	14	16	44,23	0,674	*LHCGR*
P08	13	15	139,25	0,041	*AIRE*
P09	11	39	51,48	0,092	*DAZL*
P10	16	24	63,57	1,212	*FMR1*
P11	11	25	106,59	0,144	*EIF2B4*
P12	17	33	45,10	0,169	*EIF2B4*
P14	12	39	72,46	0,010	*DACH2*
P18	12	37	76,40	0,016	*POLG*
P19	14	39	70,86	0,169	*XPNPEP2*
P20	14	39	48,30	0,092	*GDF9, POLG*
P22	13	34	51,12	1,285	*EIF2B2*
P23	11	39	140,93	0,169	*GALT*
P24	12	36	133,90	0,041	*ATM, NOBOX*
P26	12	39	44,04	0,092	*ATM, GALT, NOBOX*
P29	15	36	43,10	0,169	*GALT*
P31	11	28	55,20	0,373	*EIF2B4, GALT*
P32	11	37	65,10	0,041	*GALT*
P36	13	29	59,68	0,100	*DAZL*
P37	11	33	60,27	0,900	*HS6ST2*
P38	17	24	44,28	0,050	*HS6ST2*
P39	14	30	47,53	0,850	*HS6ST2*
P40	13	31	59,19	0,145	*EIF2B4, NOBOX*
P42	14	38	80,70	0,592	*AIRE*
P44	10	27	61,86	1,230	*GALT*
P45	12	30	80,00	0,900	*GALT*
P48	14	32	63,20	0,530	*GALT*

FSH/LH levels were measured at time of POI diagnosis.

## Discussion

**Fig. 1:**
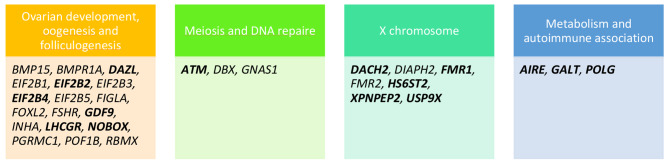
POI associated genes and their function. Genes in bold indicated that potential damaging variants or risk factors were identified in our cohort

POI is characterized by heterogenous phenotypic features with a strong genetic background that contains increasing number of genes [3]. Deleterious variants in genes related to gonadal development (oogenesis and folliculogenesis), meiosis and DNA repair, hormonal signaling, immune function, and metabolism are associated with the POI phenotype [3]. The majority of the defects we found in genes previously associated to different functions were summarised in Fig. 1.

This is the first genetic epidemiology study targeting disease genes of POI in Hungary, in which several genetic alterations were identified in POI-associated genes. In our study, we recruited 48 Hungarian patients clinically diagnosed with POI who were prescreened to *FMR1* pathogenic expansion. Targeted panel sequencing was performed to identify pathogenic variants that correspond to the list of genes known to potentially cause POI.

A monogenic defect was identified in 16.7% (8 of 48) and a potential genetic risk factor was found in 29.2% (14 of 48) and susceptible oligogenic effect was described in 12.5% (6 of 48) of women with POI using the customized targeted panel sequencing. A total of two pathogenic and 6 likely pathogenic variants were identified in 16.7% (8 of 48) of POI patients and considered a molecular genetic diagnosis of POI. Further 4 potentially damaging variants and 10 risk factors were described in 42.7% (20 of 48) of POI patients, which could potentially contribute to the disease mechanism. These variants are related to 15 genes: *AIRE* (P08 and P42), *ATM* (P24 and P26), *DACH2* (P03 and P14), *DAZL* (P09 and P36), *EIF2B2* (P22), *EIF2B4* (P11, P12, P31 and P40), *FMR1* (P10), *GALT* (P23, P26, P29, P32, P44, P45 and P48), *GDF9* (P20), *HS6ST2* (P37, P38 and P39), *LHCGR* (P05), *NOBOX* (P24, P26 and P40), *POLG* (P18 and P20), *USP9X* (P03) and *XPNPEP2* (P04 and P19). These changes included 20 missense, one frameshift and one splice site variants (Table 1. and 2.). The major concern with our results is the small sample size, which is scarce to represent the data characteristics of Hungarian population. Despite the average age of patients at the time of POI onset was 33 years, POI is recognized relatively late in some patient in Hungary. The main reason behind this observation rely the general clinical practice: if a women at a younger age experience rare or absent menstrual cycle contraceptive medication is prescribed without further clinical evaluation. Thus, amenorrhea or other hormonal problems only become apparent when the patient stops taking contraceptives and trying to conceive. According to the Hungarian Central Statistical Office (KSH), the average age at birth in Hungary is 30 years, which means that in the general population infertility problems only become apparent around the age of 30.

The frequency of variants in different POI associated genes were compared to their frequency of literature (Table 4.). Most of the observed frequencies were similar to the literature, except *EIF2B* and *GALT*. Both of these genes potential risk factor were detected which could influence the phenotype, although it is unlikely that they can be responsible for the development of the disease by themselves.

**Table 4:** Frequency of variants in different POI associated genes both in the literature and our cohort

	Frequency	
	In the literature	In our cohort
X chromosome aberrations		
Turner syndrome and related defaults	4–5%	0%
Triple X syndrome	1–4%	0%
Fragile X syndrome (*FMR1* premutation)	3–15%	0%
Gene variants (*BMP15*, *PGRMC1*, *USP9X*, *XNPEP2*, *DACH2*, *HS6ST2*)	3–13.5%	16.7%
Autosomal aberrations		
*Complex disorders*		
Galactosemia (*GALT*)	Rare	16.7%
BPES (*FOXL2*)		-
APECED (*AIRE*)		4.2%
Mitochondrial diseases (*POLG*)		4.2%
Demirhan syndrome (*BMPR1B*)		0%
PHP1a (*GNAS*)		0%
Ovarioleucodystrophy (*EIF2B*)		10.4%
Ataxia telangiectasia (*ATM*)		4.2%
Perrault syndrome (*HSD17B4*, *HARS2*, *CLPP*, *LARS2*, *C10ORF2*)		-
Premature aging syndromes: Bloom syndrome (*BLM*) Werner syndrome (*WRN*) GAPO disease (*ANTXR1*)		-
*Isolated diseases*		
FSH/LH resistency (*FSHR* and *LHCGR*)	0–1%	2.1%
*INHA* variants	0–11%	-
*GDF9* variants	1.4%	2.1%
*FOXO3* variants	2.2%	-
*NOBOX* variants	0–6%	6.3%
*FIGLA* variants	1–2%	-
*NR5A1* variants	1.6%	-
*LHX8* variants	Rare	-
DNA replication, repair and meiosis gene variants (*DAZL*, *DMC1*, *MSH4*, *MSH5*, *SPO11*, *STAG3*, *SMC1B*, *REC8*, *POF1B*, *HFM1*, *MCM8*, *MCM9*, *SYCE1*, *PSMC3IP*, *NUP107*, *FANCA*, *FANCC*, *FANCG*, *ATM*)	N/A	8.4%

The p.Ser227Leu and p.Tyr1475Cys amino acid changes were identified in *ATM* (ataxia-telangiectasia mutated gene) gene, which is required for cell-cycle checkpoint signaling pathways. It was the first DNA repair gene associated with POI [25]. *ATM* contributed to ovarian function and alterations of it results syndromic POI, characterized by primary amenorrhea. The gonads of patients with *ATM* defects are hypoplastic with germ cell deficiencies [26]. Evolutionary conservation and *in silico* predictive tools for both of these variants were unclear. In conclusion, data on these substitutions were insufficient for clear phenotype classification. Thus, the clinical significance of these aminoacid changes were uncertain.

The *EIF2B (*eukaryotic translation initiation factor 2B) genes encode the five subunits of the eukaryotic translation initiation factor 2B (*EIF2B* alpha to epsilon), which plays a role in the first step of protein synthesis. The dysfunctional *EIF2B* may be responsible for the increased apoptosis of ovarian follicles leading to POI [27]. Recently three of the five *EIF2B* genes (*EIF2B1, 2*, 3, *4* and *5*) were reportedly involved in patients who presented with POI and white matter abnormalities on MRI (ovarioleukodystrophy) [28]. The Ala127Val substitution in *EIF2B2* gene predicted to be VUS with minor pathogenic evidences which could potentially contribute to the POI phenotype even in heterozygous state, although none of the functional analysis of this residue led to certain results [18]. Thus, in women of childbearing age with mutations in *EIF2B* family genes, special attention should be paid to the possibility of POI [29].

Moreover, several other factors play crucial roles in the recruitment, development, and maturation of follicles and oocytes and mutations in genes involved in this process, such as *DAZL* (deleted in azoospermia like), may lead to the POI phenotype. *DAZL* gene is originally expressed in germ cells and essential in the beginning of meiosis and in ovarian development [13, 30]. In this study two potentially damaging variants were identified from which p.Ser288Asn was firstly described in our cohort. Both detected variants had controversial prediction scores without functional data, thus we classified them as VUS. However, heterozygous and homozygous missense substitutions in *DAZL* gene were previously identified in infertile woman associated with secondary amenorrhea [31]. Therefore, pathogenic variants were suggested to be a rare cause of male and female infertility [11]. Further studies focusing on *DAZL* function in meiotic pathway could elucidate the molecular background of human meiosis and would reveal new ways to regenerate oocytes [32].

Mutations in genes encoding fertility associated hormone receptors, such as *FSHR* and *LHCGR*, are possible contributors of ovarian functional impairment causing heterogenous clinical phenotypes [33]. The *LHCGR* (luteinizing hormone/choriogonadotropin receptor) gene, which encodes the luteinizing hormone (LH) and human chorionic gonadotropin (hCG) hormone receptors, is transcribed in granulosa cells during the last stages of the preovulatory follicles [34]. Approximately 300 polymorphisms have been reported in the *LHCGR* gene [35]. Some studies have determined that inactivating alterations in *LHCGR* gene are associated with increased LH level, enlarged ovaries, oligomenorrhea, resistance to LH or hCG hormones, and infertility [36]. On the contrary, activating mutations in affected women produce hyperandrogenism [37]. The p.Gly71Arg substitution in the luteinizing hormone receptor (LHR) ectodomain, which was detected in our cohort, was considered as an inactivating misfolding mutation. Given the unusual observation that p.Gly71Arg substitution was associated with decreased cell surface receptor expression due to intracellular retention, followed by increased efficacy for hormone stimulation [19, 20]. Because of the previous observations, p.Gly71Arg variant considered as a potential pathogenic mutation in the background of our patient’s phenotypes.

In addition, growth factors such as TGFβ family members are essential in ovarian functions [38]. The *GDF9* (Growth Differentiation Factor 9) encoded protein is a member of this previous family, which plays a crucial role in folliculogenesis [39]. Mutations of it follow autosomal dominant inheritance pattern [40]. Decreased expression and/or altered activity of the protein have been determined for most of these variants [41]. Heterozygous variants of *GDF9* have been associated with POI, decreased ovarian reserved (DOR), polycystic ovarian syndrome (PCOS) and mothers of dizygotic twins (DZT) suggesting the involvement of *GDF9* in multiple aspects of ovarian function [42]. Despite the described association of heterozygous variants with ovarian pathology, heterozygous *GDF9* variants have also been observed in healthy women [43], casting doubt on haploinsufficiency of *GDF9* causing POI. The heterozygous *GDF9* variants may be associated with a less severe phenotype (i.e. POI with secondary amenorrhea) whereas biallelic variants may lead to a more severe phenotype, such as primary amenorrhea [41]. Although, in our patient the detected p.Gly320Ser heterozygous variant with the p.Gly268Ala heterozygous substitution in *POLG* gene could deteriorate the phenotype.

In the past two decades some transcription factors, such as NOBOX protein, associated with postnatal oocyte differentiation [44]. The *NOBOX* gene (newborn ovary homeobox) is expressed in primordial germ cells, oocytes and granulosa cells [45]. *NOBOX* directly controls several ovarian genes, including the previously mentioned *GDF9* [46]. These data highlight that *NOBOX* plays a cardinal role in folliculogenesis [47]. Initially, heterozygous variants with dominant negative effects were identified [44], and loss-of-function mutations were reported with a 6.2% prevalence in a POI cohort [48]. All of the three detected variants associated with a second mutation in a different gene, which underline the potential oligogenic effects in the background of POI phenotype. We suppose a potential modifying effects of these variants, but further functional studies are needed to clarify them.

Both *DACH2* (Dachshung homolog 2) and *XPNPEP2* (X-prolyl aminopeptidase 2) genes were previously associated with POI phenotypes [23]. These genes may be required in double dose throughout the life of the oocytes, when the presence of two active X chromosomes is a rule. Moreover, previous results suggested that *XPNPEP2* gene, which encodes an Xaa-Pro aminopeptidase with an unknown substrate, at least partially rescues from X inactivation [49]. Recent studies indicated that variants of *DACH2* gene might serve as a genetic risk factor for POI by modifying the normal differentiation of ovarian follicle [23]. Previous data suggested that p.Pro36Leu missense mutation in *DACH2* gene (P03) were more frequent in POI patients than in controls, but no significant association with POI was confirmed [23]. However, *DACH2* mutations may be extremely rare, they should be involved in the diagnostic pipeline with other risk factors, such as *FMR1* premutation analysis [23]. The *USP9X* (Ubiquitin-Specific Protease 9, X-linked) gene also rescues X inactivation and is found in a region (Xp11.4) dedicated to ovarian development [50]. *USP9X* catalyze the deubiquination of specific substrates and previous studies referred an essential conserved function of *USP9X* manifested in human folliculogenesis [13].

The *FMR1* gene, member of the fragile X-related gene family, is responsible for fragile X syndrome (FXS). An *in silico* prediction analysis suggested that 31.66% of the *FMR1* gene SNVs were disease related and that 50% of SNVs had a pathogenic effect [24]. The results of a previous structural and functional analysis revealed that p.Lys273Arg substitution did not seem to have a damaging effect on the protein, although the contribution to the disease phenotype could not be ruled out [24]. A bioinformatic analysis of the breakpoint regions identified putative candidate gene, *HS6ST2* for ovarian failure that were involved in the translocation event and its function with a literature review revealed a potential connection to the POI phenotype [51]. The detected p. Ser49Leu SNV was firstly described in our article.

Recently, mutations in the catalytic subunit of mitochondrial DNA polymerase gamma (*POLG*) were shown to segregate with POI in families with progressive external ophthalmoplegia (PEO) and multiple large-scale rearrangements of mitochondrial DNA (mtDNA) [52]. The detected p.Gly268Ala substitution was previously identified in patients with PEO with conflicting interpretation of pathogenicity [53]. Further studies are needed to confirm the connection between these variants and the POI phenotype.

The *AIRE* (autoimmune regulator) gene encoded protein main function is to regulate the clonal deletion of autoreactive T-cells. The loss of function mutations of it are responsible for polyendocrinopathies (APS I–III) [54], although variants of this gene also could cause POI associated syndromes [17]. Interestingly, both mutations are located in functional regions of the protein (p.Thr441Met in the PHD2 domain that is essential for transcriptional activation and p.Val301Met in the PHD1 domain which is involved in protein–protein interaction such as assembling the transcription-activating machinery) [55]. Basically all missense alterations in the PHD1 domain, including p.Val301Met, displayed a dominant-negative effect on *AIRE*-dependent genes and this variant is associated with POI phenotype in heterozygous state [56]. Further studies revealed that p.Val301Met alteration strongly reduced *AIRE* target gene activation in vivo generating clear differences in *AIRE* interactome compared to the wild-type protein [57]. The detected p.Thr441Met missense variant was suggested to be probably damaging by a previous functional study [22].

One of the four enzymes involved in the main galactose metabolism pathway (known as the Leloir pathway) is the *GALT* (galactose-1-phosphate uridylyltransferase) gene encoded protein. Deficiency in *GALT* causes classical galactosaemia Type 1 in autosomal dominant form, which resulted POI as the most common long-term complication [58]. Although, the possible effects of single heterozygous variants is still a question of debate as several examples exist in the literature that even a heterozygous variant could be considered as a risk factor for developing certain phenotypes in an autosomal recessively inherited gene. One of the most common variants in GALT gene with a frequency ranging from 1 to 13% is p.N314D such as in our cohort [59].

## Conclusions

High-throughput techniques have been crucial for revealing new variants both in genes which were previously associated to POI and in new candidate genes. The encoded proteins mainly play roles in gonadal development (oogenesis and folliculogenesis), meiosis and DNA repair, hormonal signaling, immune function, and metabolism. Due to the resting state of oocytes, alterations in genes involved in meiosis and DNA repair may induce different phenotypes of ovarian insufficiency, as demonstrated in various animal models [60]. POI is a very heterogeneous disorder that can be caused by a variety of factors suggesting an unknown number of variants in the background of the phenotype. Increasing number of researches using NGS pave the way to discover several disease-specific genes and variants which presented a potential association with POI features. Thus, NGS approach was recommended as a powerful tool for identifying the genetic cause of POI and can contribute to understanding the disease etiology for future diagnostic/prognostic purposes. In order to further elucidate the genetic background of POI, efforts are needed to understand the complex mechanisms by using integrated databases and approaches to predict the combination of disease-associated variants. Thus, in the future it is essential to integrate and systematically manage and verify data collected from a number of NGS studies specialized to POI patients.

## Data Availability

The raw data supporting the conclusions of this manuscript will be made available by the authors, without undue reservation, to any qualified researcher.

## Abbreviations

AA: amino acid change
aCGH: Array Comparative Genomic Hybridization
ACMG: American College of Medical Genetics
CADD: Combined Annotation Dependent Depletion
cDNA: complementary DNA
CNV: copy number variations
DNA: Deoxyribonucleic acid
emPCR: Emulsion polymerase chain reaction
FSH: Follicle-stimulating hormone
FXS: fragile X syndrome
GERP: Genomic Evolutionary Rate Profilin
IGV: Integrative Genomics Viewer
ISPs: Ion Sphere Particles
LH: Luteinizing hormone
MAF: minor allele frequency
mtDNA: mitochondrial DNA
NGS: next-generation sequencing
P/B: pathogenic/benign prediction
PCR: polymerase chain reaction
PEO: progressive external ophthalmoplegia
PHRED: Phil’s Read Editor (University of Washington Genome Center)
POF: premature ovarian failure
POI: Premature ovarian insuffiency
RP-PCR: repeat-primed polymerase chain reaction
SIFT: Scale Invariant Feature Transform
SNV: single nucleotide variant
VEST: variant effect scoring tool
VUS: variant with unknown significance
